# Comparison of Open Versus Minimally Invasive Repair of Colovesical Fistula: A Case Report and Propensity-Matched National Database Analysis

**DOI:** 10.3390/jcm14176065

**Published:** 2025-08-27

**Authors:** Alexis Volkert, Anmol Nigam, David Stover, Pravin Meshram, Rubeena Naaz, Chidiebere Onongaya, Sean Huu-Tien Nguyen, Jordan Sauve, Wolfgang Gaertner, James V. Harmon Jr.

**Affiliations:** 1 Department of Surgery, University of Minnesota, Minneapolis, MN 55455, USAharm0031@umn.edu (J.V.H.J.); 2 Department of Surgery, Astera Health, Wadena, MN 56482, USA; 3 Shadan Institute of Medical Sciences, Hyderabad 500086, India; 4 Division of Colon and Rectal Surgery, Department of Surgery, University of Minnesota, Minneapolis, MN 55455, USA

**Keywords:** colovesical fistula, enterovesical fistula, minimally invasive surgery, open surgery, diverticulitis, national inpatient sample, propensity score matching

## Abstract

**Background:** Colovesical fistulas are abnormal communications between the colon and urinary bladder, most commonly caused by diverticular disease. Although colovesical fistulas are rare, they should be suspected in patients presenting with recurrent urinary tract infections, pneumaturia, or fecaluria. We integrated two case reports with a retrospective national cohort analysis to assess the surgical treatment of colovesical fistulas. **Methods:** We report two cases of colovesical fistulas, both secondary to sigmoid diverticulitis, treated surgically via minimally invasive approaches. A retrospective analysis using the National Inpatient Sample database from 2016 to 2022 was conducted to compare outcomes of open surgery with those of minimally invasive surgery. Propensity score matching and multivariable regression analyses were used to evaluate clinical outcomes. **Results:** The first patient underwent hand-assisted laparoscopic sigmoidectomy with fistula takedown and has remained asymptomatic at 8 months, while the second patient underwent robotic-assisted sigmoidectomy with staged ileostomy reversal and has remained asymptomatic at 1 month. National data analysis showed no significant difference in mortality (<1% versus <1%, *p* = 0.931), wound complications (1.4% versus 1.0%; *p* = 0.554), or postoperative sepsis or shock (7.1% versus 5.6%; *p* = 0.114) between open and minimally invasive surgical approaches. However, the minimally invasive surgery group had significantly shorter length of stay than the open surgery group (6.9 versus 7.3 days, *p* < 0.001). **Conclusions:** Minimally invasive repair of colovesical fistulas was associated with shorter hospital stays than open surgery, with no significant differences in major complications. Early identification and timely surgical management are critical for achieving favorable outcomes.

## 1. Introduction

The most common cause of colovesical fistulas is colonic diverticular disease, accounting for approximately two-thirds of all cases [1]. Other etiologies include malignancies, iatrogenic injuries, pelvic radiation, and inflammatory bowel disease [2]. Among individuals aged >60 years, an estimated 50% have diverticulosis, and the incidence of diverticulitis continues to rise in the United States Midwest population [3]. As the prevalence of colonic diverticulitis increases, the occurrence of colovesical fistulas is also expected to become more common [4].

The diagnosis of colovesical fistula is primarily clinical and should be supported by imaging studies [5]. However, radiologic confirmation can be challenging, as demonstrated in one of the present cases. A combination of pneumaturia, recurrent urinary tract infections (UTIs), and fecaluria is usually sufficient to raise clinical suspicion [6]. Although not always definitive, cross-sectional imaging may reveal inflammatory changes, a mass, or the fistula tract [7]. Colonoscopy is typically recommended to rule out malignancy; however, it is rarely diagnostic for fistulas [8]. Moreover, cystoscopy is considered when bladder pathology, particularly malignancy, is suspected on imaging [9].

Here, we present two cases of colovesical fistula secondary to diverticulitis: the first managed with a hand-assisted laparoscopic technique and the second with a robot-assisted laparoscopic approach. This study also aimed to describe the clinical presentations, diagnostic approaches, surgical management, and postoperative complications that can occur during colovesical fistula repair. A literature review and national database analysis were conducted to compare patient demographics and postoperative outcomes between open and minimally invasive surgical approaches for colovesical fistula repair. A total of 2765 patients hospitalized with the diagnostic code enterovesical fistula (EVF) were included using the national database analysis. Data were extracted covering the years 2016–2022. A 1:1 propensity score-matched analysis was conducted comparing patients who underwent open colovesical fistula repair (*n* = 1378) with those who underwent minimally invasive repair (*n* = 1378). Their outcomes were matched and compared using standard mean differences to evaluate postoperative outcomes between the two surgical approaches. To our knowledge, this is the first study to integrate detailed case reports, a propensity-matched national database analysis, and a focused literature review to comprehensively evaluate colovesical fistula management approaches.

## 2. Materials and Methods

### 2.1. Case Report Identification

A retrospective chart review was performed to obtain two representative case reports of colovesical fistula from our clinical practice. Both patients underwent surgical intervention for colovesical fistulas secondary to sigmoid diverticulitis. Relevant clinical data were extracted, including presenting symptoms, diagnostic imaging, operative findings, and postoperative outcomes.

### 2.2. National Inpatient Sample Data Extraction

A retrospective cohort analysis was performed using data from the National Inpatient Sample (NIS) database, covering the years 2016–2022. The study period began in 2016 to ensure complete and consistent use of International Classification of Diseases, 10th Revision (ICD-10) codes, which were nationally implemented in October 2015. Data for 2023 and 2024 were excluded due to limitations in the NIS dataset availability, which currently extends only through 2022.

Extracted variables included patient demographics (age, sex, race/ethnicity), comorbidities, diagnosis codes, procedure codes, length of stay, total hospital charges, discharge disposition, in-hospital mortality, and hospital characteristics. Hospital characteristics in the dataset included region, bed size, teaching status, and ownership. Hospital bed size is a variable that categorized hospitals as small, medium, or large based using the HCUP definition. This definition was based on bed count, adjusted for region, urban/rural location, and teaching status (e.g., small: 1–49 beds at rural institutions in the Northeast, large: 450+ beds at urban teaching institutions in the South) [10]. All variables were extracted from the NIS Core, Hospital, and Severity files.

Primary outcomes were in-hospital mortality, postoperative rates of sepsis or shock, and rates of wound complications. Secondary outcomes were length of stay, total hospital charges, and hospital bed size.

The analysis included adult patients (aged ≥ 18 years) hospitalized with a diagnosis code for EVF. Obstetric-related fistulas (e.g., vesicouterine fistulas) and patients under 18 years of age were excluded.

### 2.3. Propensity Score Matching and Statistical Analysis

A 1:1 propensity score-matched analysis was conducted to compare patients who underwent a minimally invasive surgical approach with those who underwent open surgery. Propensity scores were calculated using logistic regression models incorporating relevant demographic, clinical, and hospital-level covariates. Postmatching balance was assessed using standardized mean differences.

Before conducting inferential analyses, we examined the distribution of continuous variables through visual inspection of histograms and Q–Q plots, along with descriptive statistics including skewness and kurtosis [11]. Given the large sample size in the NIS, formal normality tests (e.g., the Shapiro–Wilk test) were not relied upon, as they are highly sensitive to minor deviations from normality [12]. Length of hospital stay and total charges demonstrated marked right-skewness; therefore, generalized linear models with a gamma distribution and log link were applied to account for the skewed distribution of these continuous outcomes [13]. For multivariable modeling, logistic regression was used for categorical outcomes, including mortality and postoperative complications. All analyses adjusted for residual confounders.

Statistical analyses were performed using R version 4.5.0 (R Foundation for Statistical Computing, Vienna, Austria) and IBM SPSS Statistics for Windows, Version 28.0.1.1 (IBM Corp., Armonk, NY, USA). Missing data were minimal and handled via available case analysis. No data imputation methods were applied. A two-sided *p*-value of <0.05 was considered statistically significant.

### 2.4. Ethical Considerations

The NIS is a publicly available, de-identified dataset; therefore, this study was exempt from Institutional Review Board (IRB) approval. Case reports were obtained under institutional policy, which does not require IRB approval at the University of Minnesota.

## 3. Case Presentation

### 3.1. Case 1

A 59-year-old male patient presented with a 2-week history of dysuria, cramping abdominal pain, hematuria, and pneumaturia. He had experienced two previous UTIs, each treated with a 10-day course of antibiotics, which led to transient symptom relief. The patient denied a history of diverticulitis, with a remote laparoscopic cholecystectomy being his only prior surgery. Two 5 mm tubular adenomas were removed via endoscopic polypectomy 5 years ago. Numerous diverticula were noted in the sigmoid colon.

The abdomen was soft on physical examination, with mild tenderness in the lower quadrants. Urinalysis revealed >50 white blood cells (WBCs; normal range: 0–5 high-power field [HPF]), 5 red blood cells (RBCs; normal range: 0–2 HPF), and moderate bacteria. The initial urine culture was negative; however, the second culture yielded 10,000 colonies of

*Lactobacillus*. Peripheral blood counts were within normal limits, including the WBCs (6.5 × 109 L; normal range: 4.8–10.8 µL) and C-reactive protein (CRP < 0.30 mg/dL; normal range: 0.00–0.50 mg/dL).

**Figure 1:**
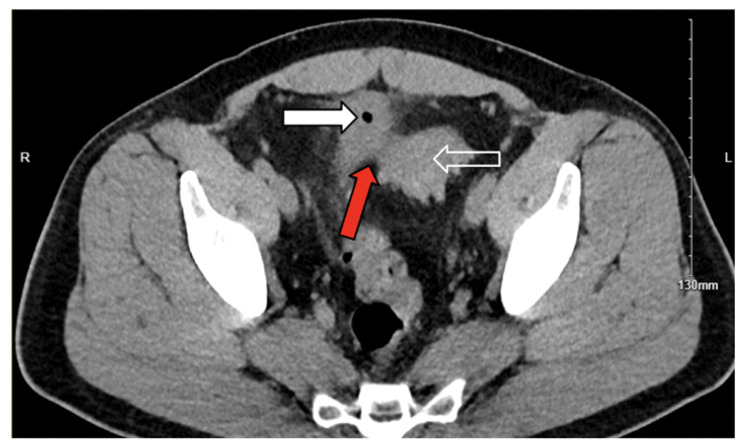
Axial noncontrast computed tomography image demonstrating confluent fat stranding and inflammation (red arrow) between the sigmoid colon (white outlined arrow) and left superior bladder wall. White arrow: air within bladder.

**Figure 2:**
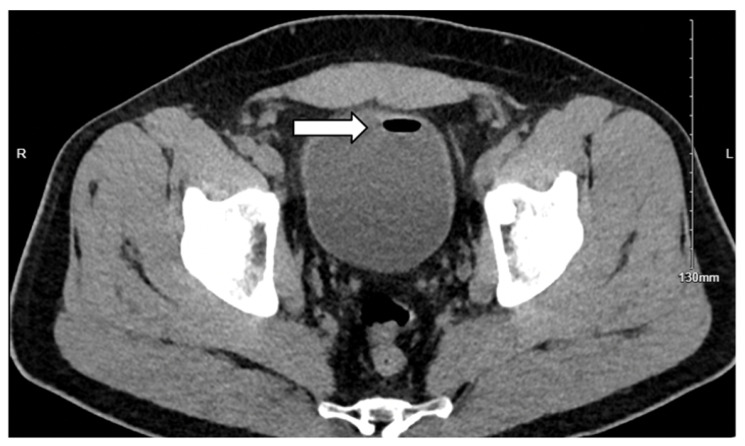
Axial noncontrast computed tomography image demonstrating the presence of air within the bladder (white arrow).

**Figure 3:**
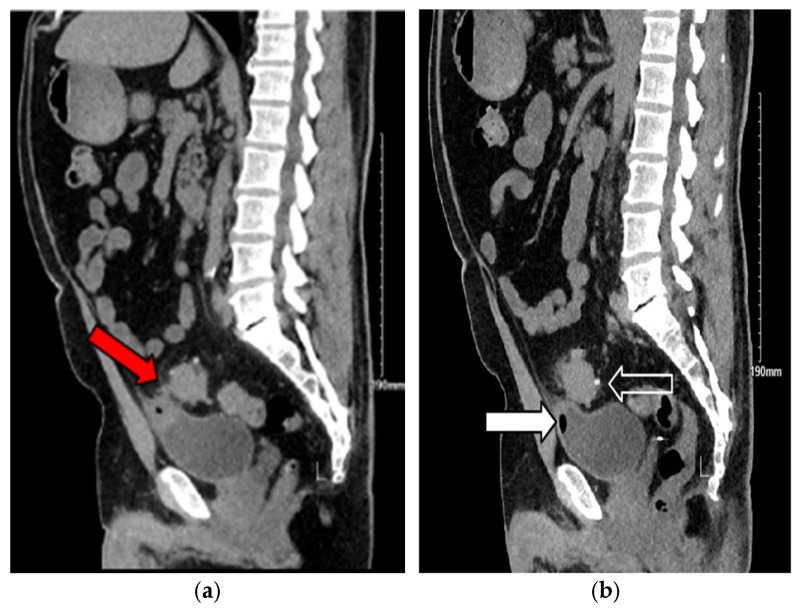
(**a**) Sagittal noncontrast computed tomography (CT) image showing air within the bladder and sigmoid diverticulitis with a suspected fistulous tract between the sigmoid colon and bladder (red arrow). (**b**) Sagittal noncontrast CT image demonstrating diverticular outpouching of the sigmoid colon (white outlined arrow) and presence of air within the bladder (white arrow), consistent with colovesical fistula.

**Figure 4:**
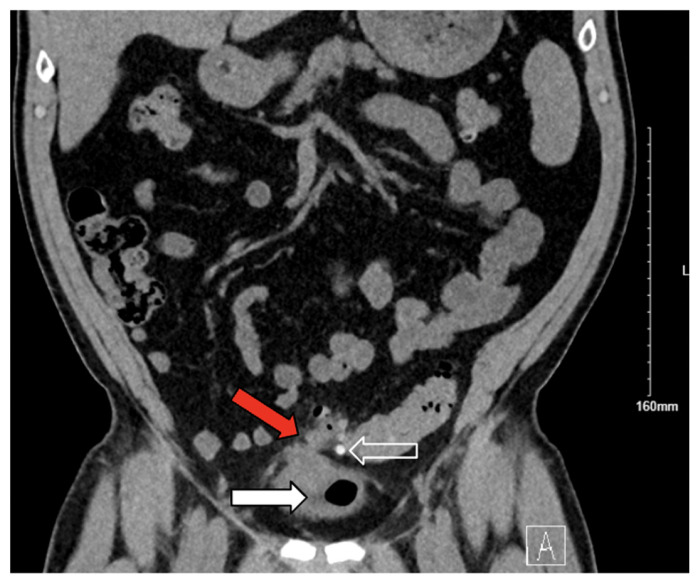
Coronal noncontrast computed tomography showing air within the bladder (white arrow) and superior sigmoid colon diverticulitis (white outlined arrow). A suspected fistulous connection between the sigmoid colon and bladder is indicated (red arrow), consistent with colovesical fistula.

Abdominal computed tomography (CT) demonstrated sigmoid diverticulitis, fat stranding, and close apposition of the sigmoid colon and urinary bladder (Figure 1). The bladder walls appeared thickened, and gas was identified within the urinary bladder (Figure 2). Although a colovesical fistula was suspected, the tract was not visualized on imaging (Figure 3a,b and Figure 4).

**Figure 5:**
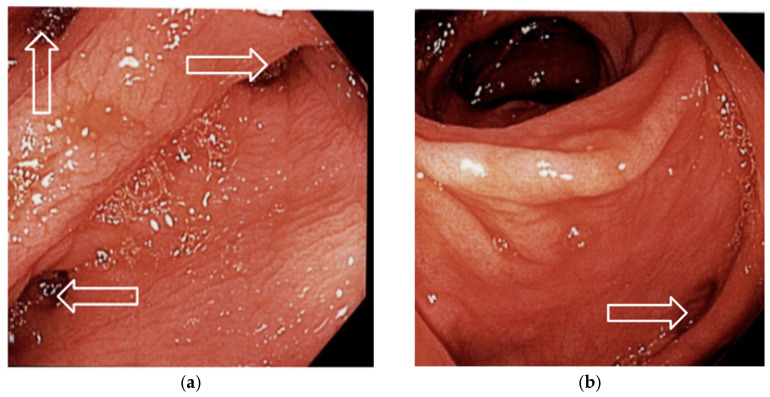
(**a**,**b**) Colonoscopy images showing multiple diverticula (white outlined arrows) in the sigmoid colon, consistent with diverticular disease.

Urology consultation with flexible cystoscopy revealed a small fistula tract on the posterior bladder wall. No bladder diverticula or neoplastic lesions were identified. Colonoscopy was performed to rule out malignancy and showed no additional abnormalities (Figure 5a,b). The patient consented to undergo hand-assisted laparoscopic repair of the colovesical fistula.

Intraoperatively, dense adhesions, scarring, and granulation tissue were noted at the fistula site on the bladder dome. A segment of the sigmoid colon was embedded within the bladder. En bloc resection of a small ellipse of the bladder wall and the involved sigmoid segment was performed. The bladder wall was re-approximated in the following two layers: the mucosal layer with poliglecaprone 25 (Monocryl^®^ 3-0, Ethicon, Inc., Somerville, NJ, USA) and muscular layer with polydioxanone sutures (PDS II 2-0, Ethicon, Inc., Somerville, NJ, USA). A side-to-side sigmoid colon stapled anastomosis was completed extracorporeally via a hand-assisted port. The abdominal cavity was irrigated, and hemostasis was achieved using electrocautery. A 19-Fr Blake (Ethicon, Inc., Somerville, NJ, USA) drains were placed in the pelvis near the bladder repair site. The fascia was closed with polydioxanone sutures (PDS II 0-looped, Ethicon, Inc., Somerville, NJ, USA), and the skin was re-approximated with surgical staples.

**Figure 6:**
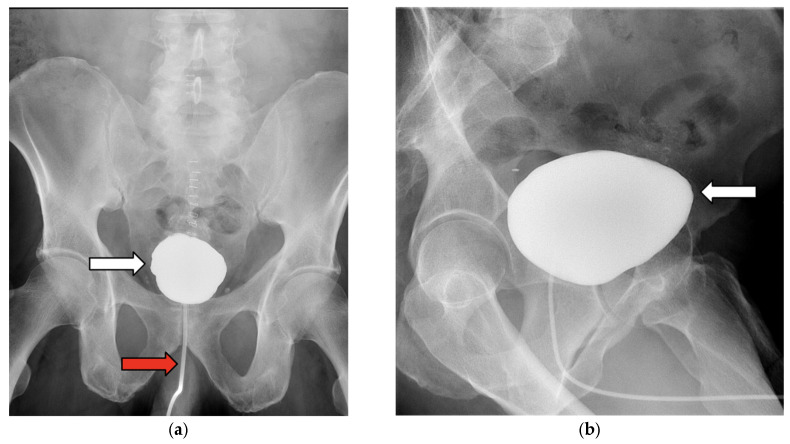
(**a**,**b**) Postoperative cystogram obtained 2 weeks after surgical repair, demonstrating complete resolution of the colovesical fistula without evidence of extravasation. The bladder is outlined (white arrows), and a Foley catheter is visualized (red arrow).

The patient’s diet was advanced after the return of bowel function, and he was discharged on postoperative day (POD) 5. A cystogram performed 2 weeks postoperatively demonstrated complete healing without complications (Figure 6a,b). The patient remained asymptomatic and complication-free at his latest 8-month follow-up.

### 3.2. Case 2

A 45-year-old male with no significant medical history presented with a 10-day history of intermittent dysuria, dark-brown urine, urinary debris, and dull right mid-back pain. Although not initially recognized, these symptoms were ultimately attributed to an EVF. The patient denied systemic or gastrointestinal symptoms and had no history of diverticulitis, malignancy, inflammatory bowel disease, or colonoscopy. Initial urinalysis showed 11–20 WBCs (normal range: 0–5 HPF), 0–2 RBCs (normal range: 0–2 HPF), and occasional bacteria. Peripheral blood counts were within normal limits, including WBCs (6.4 × 10^9^ L; normal range: 4.0–11.0 µL). The patient was empirically treated for a UTI; however, antibiotics were discontinued following a negative culture result. The treatment was later resumed due to persistent symptoms.

**Figure 7:**
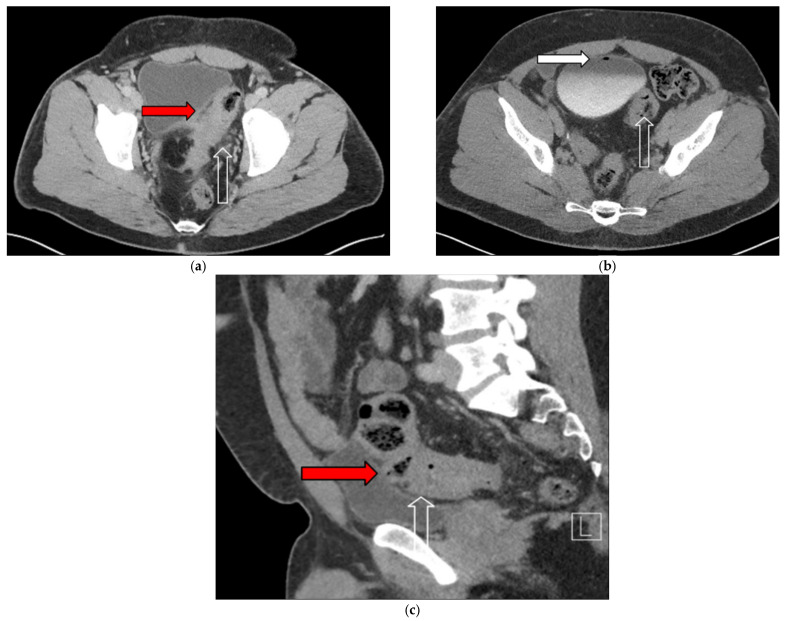
(**a**–**c**) Contrast-enhanced computed tomography images demonstrating inflammatory changes involving the sigmoid colon and bladder wall (red arrows), suggestive of colovesical fistula: (**a**,**c**) show inflammatory stranding between the sigmoid colon and bladder wall and (**b**) shows intravesical air (white arrow). Sigmoid colon with thickening consistent with diverticulitis is noted in all panels (white outlined arrows).

Two months later, the patient reported malodorous urinary debris and experiencing two episodes of hematochezia. Cystoscopy revealed the presence of fecal material within the bladder, raising suspicion of a colovesical fistula. Cross-sectional imaging confirmed the presence of a fistulous tract between the sigmoid colon and bladder (Figure 7). Although a poppy seed test was suggested to support this diagnosis, no further documentation was available.

The patient was referred for definitive colorectal surgery. Preoperative colonoscopy showed a benign rectal lesion suggestive of fistula formation; however, further advancement was hindered by sigmoid inflammation and tortuosity. Approximately 10 months after symptom onset, he underwent robotic-assisted laparoscopic sigmoidectomy with fistula takedown, ureteral stent placement, and flexible sigmoidoscopy.

Intraoperatively, extensive inflammation and dense adhesions were observed, along with two fistulas: a colovesical fistula involving the mid-to-upper bladder and a rectourethral fistula located 2 cm proximal to the dentate line. These findings indicated a more extensive pelvic disease than initially suspected. The bladder defect was repaired, and a hand-sewn coloanal anastomosis was performed using polyglactin 910 (Vicryl^®^ 2-0 and 3-0, Ethicon, Inc., Somerville, NJ, USA). Indocyanine green angiography and intraoperative endoscopy confirmed adequate perfusion and an intact anastomosis, respectively. Frozen sections were negative for malignancy. A silicone closed-suction drain (Jackson–Pratt^®^, Cardinal Health, Dublin, OH, USA) and an omental pedicle flap were placed at the rectourethral repair site, and a diverting loop ileostomy was created. The skin was closed with polydioxanone (PDS II^®^ 0, Ethicon, Inc., Somerville, NJ, USA) and poliglecaprone 25 (Monocryl^®^ 4-0, Ethicon, Inc., Somerville, NJ, USA). Finally, the patient was discharged on POD 3 with a Foley catheter and a drain in place.

**Figure 8:**
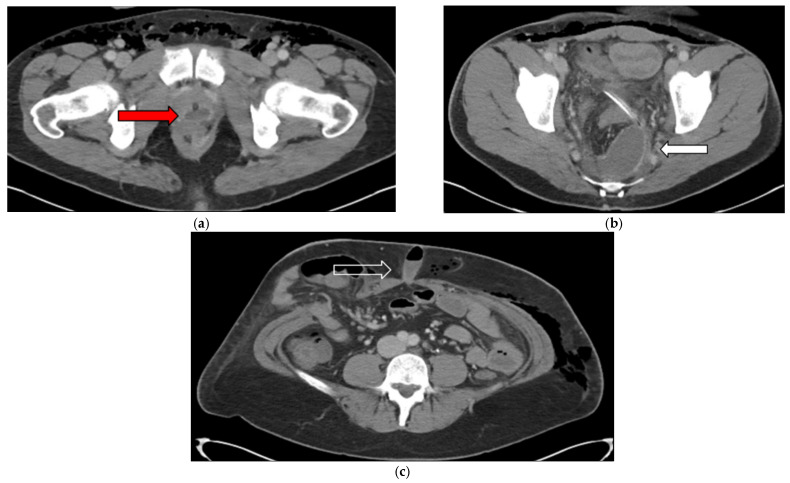
(**a**–**c**) Postoperative contrast-enhanced computed tomography images demonstrating multiple fluid collections. (**a**) Fluid collection posterior to the prostate, measuring 2.9 cm (red arrow). (**b**) Fluid collection in the left presacral region, measuring 6.2 cm (white arrow). An operative closed-suction drain is also visible. (**c**) Fluid and gas collection at the umbilicus, measuring 4.2 cm (white outlined arrow).

The patient’s postoperative course was complicated by intra-abdominal and subcutaneous fluid collections (Figure 8), necessitating readmission for intravenous antibiotics and image-guided drainage. His condition improved with treatment, and he was treated with a 2-week course of oral antibiotics.

**Figure 9:**
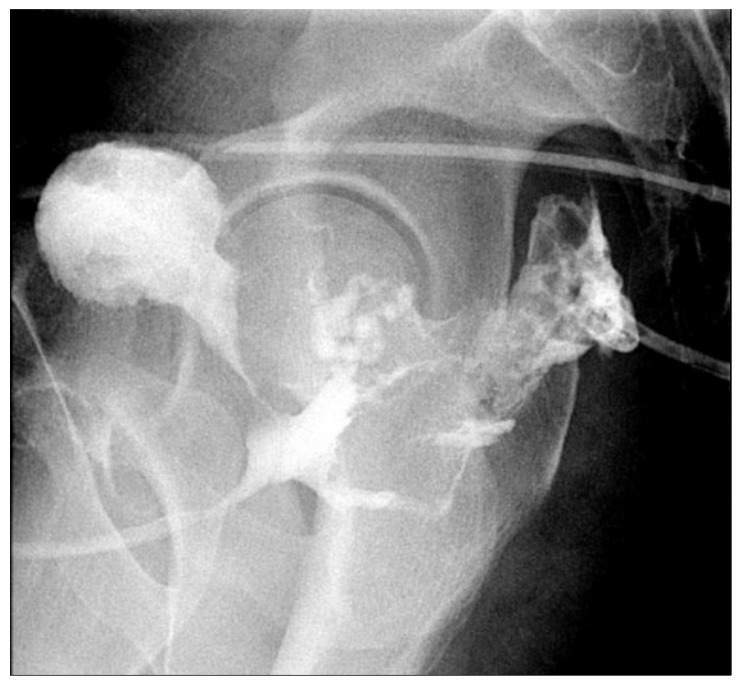
Postoperative cystogram demonstrating extraluminal contrast extravasation from a prostatic urethral defect, extending into a presacral fluid collection containing a pigtail drain.

Repeat imaging showed a reduction in the fluid collections after the antibiotic course was completed. However, urethrography revealed a persistent prostatic urethral leak (Figure 9). Flexible sigmoidoscopy showed a small coloanal anastomotic dehiscence, while cystoscopy identified a residual urethral tract. Persistent presacral collection and minimal urethral contrast leakage were noted at 3 months postoperatively. This was ultimately attributed to a persistent sinus tract at the anastomosis communicating with the presacral collection via the rectum. Intra-abdominal drains and a Foley were maintained and subsequently removed after endoscopy confirmed closure of the prostatic end of the fistula tract. The sinus tract would ultimately require surgical repair.

**Figure 10:**
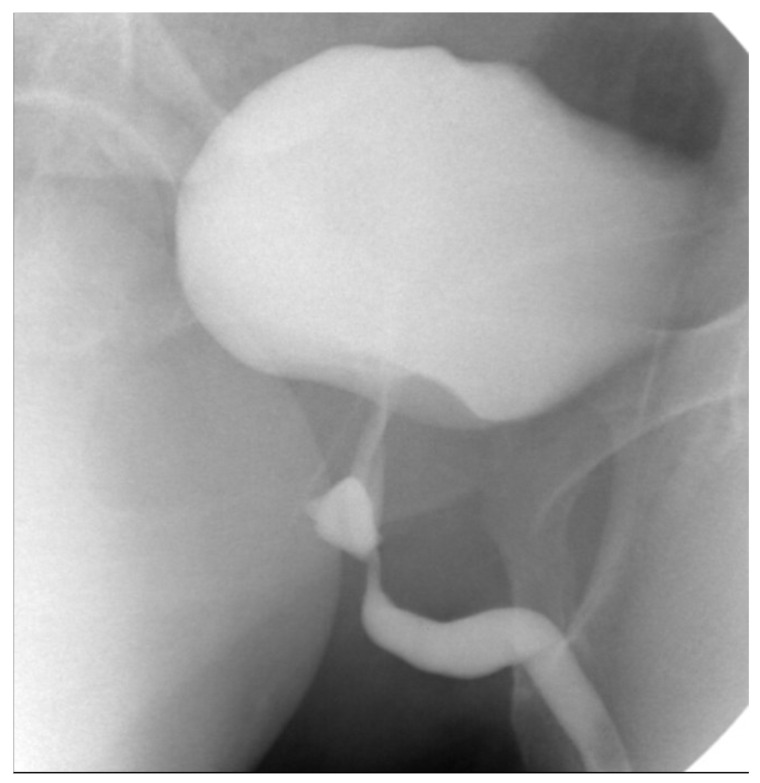
Interval cystogram demonstrating resolution of the previously identified fistulous tract, with no evidence of contrast extravasation.

Follow-up evaluations using cystography (Figure 10) and endoscopy showed no residual fistulous communication. However, CT imaging at 4 months demonstrated contrast extravasation from the coloanal anastomosis into the presacral space, without bladder or urethral involvement. The patient subsequently underwent surgical closure of the anastomotic sinus tract, which involved curettage, debridement, and irrigation, followed by closure with poliglecaprone 25 (STRATAFIX™ Spiral Monocryl^®^ 3-0, Ethicon, Inc., Somerville, NJ, USA). The following month, he underwent takedown of the diverting ileostomy in standard fashion, with closures using poliglecaprone 25 (Monocryl^®^ 4-0), silk (Silk 4-0), polydioxanone (PDS II^®^ #1), and polyglactin 910 (Vicryl^®^ 2-0) sutures (all Ethicon, Inc., Somerville, NJ, USA).

The patient recovered without complications following the second procedure and was discharged on POD 4. At 1-month follow-up, he remained clinically well, with no urinary or gastrointestinal complaints, tolerating a regular diet, and had resumed normal activities. This case highlights the diagnostic complexity and necessity of a multidisciplinary, staged surgical approach for managing complex enterovesical and rectourethral fistulas.

## 4. Results

### 4.1. Patient Selection and Matching

Overall, 2756 patients diagnosed with EVF were identified from the NIS database between 2016 and 2022. After applying 1:1 propensity score matching, 1378 patients were included in each treatment group: those who underwent minimally invasive surgery (MIS) and those who underwent open surgery. Postmatching covariate balance was achieved across relevant clinical and hospital-level variables.

### 4.2. Postoperative Outcomes

Table 1 presents the postoperative outcomes for both groups. Appendix A provides additional variables associated with undergoing MIS versus open surgery for colovesical fistula repair. No significant differences were found in in-hospital mortality between the MIS and open surgery groups (0.4% versus 0.7%; adjusted odds ratio [OR]: 1.087; 95% confidence interval [CI]: 0.164–7.207; *p* = 0.931). Wound complications (1.0% versus 1.4%; OR: 0.799; 95% CI: 0.380–1.680; *p* = 0.554), postoperative pneumonia (1.5% versus 1.4%; OR: 1.249; 95% CI: 0.627–2.487; *p* = 0.528), and sepsis or shock (5.6% versus 7.1%; OR: 0.740; 95% CI: 0.509–1.075; *p* = 0.114) were also not significantly different between the groups. Similarly, postoperative hemorrhagic shock occurred at comparable rates (1.1% versus 1.0%; OR: 1.065; 95% CI: 0.500–2.268; *p* = 0.870).

### 4.3. Length of Stay and Hospital Charges

Patients undergoing MIS had a significantly shorter hospital stay than those undergoing open surgery (mean: 6.89 versus 7.49 days; OR: 1.11; 95% CI: 1.06–1.15; *p* < 0.001). However, no significant difference in total hospital charges was found between the two groups (USD 124,672 versus 129,102; OR: 0.995; 95% CI: 0.95–1.04; *p* = 0.82).

### 4.4. Hospital Characteristics

Hospital bed size distribution showed that large hospitals managed the majority of cases in both groups. A significantly higher proportion of MIS procedures was performed compared to open surgeries in small hospitals (21.7% versus 18.9%; OR: 1.325; 95% CI: 1.068–1.644; *p* = 0.011), while the distribution among medium and large hospitals was not statistically different.

## 5. Discussion

Colovesical fistulas are rare complications, most commonly arising from complicated sigmoid diverticulitis [14]. In a review of the NIS, Underhill et al. reported that 0.05% of all hospital discharges related to diverticular disease involved diverticular fistulas [15]. Diverticular disease accounts for up to 80% of EVFs, with the majority being colovesical [16]. Malignant colonic neoplasia is the second most common cause of EVFs, contributing to up to 20% of cases, whereas Crohn’s disease is responsible for approximately 7% of cases [17,18]. The same study also reported a higher incidence of colovesical fistulas in men (*n* = 1100) than in women (*n* = 856). However, overall diverticular fistulas of all types were more common in women (*n* = 2200) than in men (*n* = 1643), likely due to the inclusion of obstetric and genitourinary fistulas. A smaller cohort of 49 patients similarly showed a male predominance, with 42 and 7 cases in men and women, respectively [19]. This sex difference is believed to result from the anatomic interposition of the uterus between the bladder and colon, which may lower the risk of colovesical fistula formation in women [20].

Diagnosing colovesical fistulas remains challenging due to their clinical resemblance to UTIs [21]. The rarity of this condition further contributes to its under-recognition [22]. A 6-year single-center series of 90 patients with colovesical fistulas reported the most common presenting symptoms to be pneumaturia (90%), fecaluria (76%), abdominal pain (71%), recurrent UTIs (66%), hematuria (30%), localized peritonitis (14%), sepsis (9%), and abdominal mass (9%) [17,23]. In the present case, a 59-year-old male patient without benign prostatic hyperplasia or recent urologic intervention presented with recurrent symptoms consistent with a fistula.

The optimal diagnostic modality for colovesical fistulas is still debated. Contrast-enhanced CT is the most frequently used tool and is recommended by the American College of Radiology as the first-line modality, with reported sensitivity of 60–94.1% [24,25]. CT was performed without contrast in the first case due to the patient’s allergy to contrast. Other diagnostic tests include colonoscopy, cystoscopy, magnetic resonance imaging, and water-soluble contrast enema (diatrizoate meglumine and diatrizoate sodium; commonly known by the brand name Gastrografin) [25]. Identifying the underlying pathology that contributes to fistula formation is crucial, as it influences the surgical approach. Since the most common etiology of colovesical fistulas is diverticular disease, followed by malignancies, colonoscopy and cystoscopy—although limited in sensitivity—are necessary to exclude malignancy. A cohort study of 49 patients found that colonoscopy and cystoscopy detected the fistula in only 8% and 10% of cases, respectively. However, both tests remain essential for evaluating neoplastic causes [26].

An emerging diagnostic method is the “poppy seed test”, which demonstrated up to 100% sensitivity in a pooled analysis of 57 patients [19,27]. In this test, patients ingest 50 mg of poppy seeds with 12 and 6 ounces of liquid or yogurt, respectively, and their urine is monitored for up to 48 h for seed passage [28]. A positive result strongly suggests a colovesical fistula. While the test is cost-effective and outperforms CT imaging for diagnosing colovesical fistulas [27], it cannot localize the colovesical fistula or rule out malignancy.

Surgical repair is the primary treatment for colovesical fistula. A systematic literature review of 1061 patients concluded that one-stage colonic resection with primary anastomosis is the most commonly performed and preferred treatment approach for colovesical fistula [3]. Previous studies have found no significant difference in complication rates between primary anastomosis and staged operations [26]. Nonetheless, Hartmann’s procedure or a staged approach may be warranted in patients with severe diverticulitis, abscess formation, sepsis, or large bowel obstruction secondary to a stricture or mass. Reported postoperative complication rates vary from 6% to 49% [19]. A recent retrospective cohort study of 512 patients undergoing surgery for colovesical fistulas reported a 30-day postoperative complication rate of 30% [29]. The most common postoperative complications included surgical site infections, bleeding, and sepsis. Lower preoperative hematocrit level and age >65 years have been associated with increased risk of complications. A retrospective study compared the use of polydioxanone (PDS II) versus triclosan-coated polydioxanone (PDS Plus) and the incidence of surgical site infections for hypospadias repair. The abovementioned study found that the use of PDS Plus in patients resulted in a significantly lower number of surgical site infections than the use of PDS II (*n* = 18 (6.9%) versus *n* = 4 (1.4%), *p* < 0.001) [30]. PDS II was used in both of our cases; however, the future use of PDS Plus could be considered during colovesical fistula repairs since surgical site infection is the leading postoperative complication, as previously stated.

The key principle in the operative management for colovesical fistulas is segmental resection of the diseased colon [31]. Historically, attempts to repair the fistula without resecting the affected colon have usually been reserved for patients who are not surgical candidates or have terminal conditions [32]. In one cohort study of 50 patients, six declined surgery [33]. Although no statistically significant difference was found in mortality, patients managed without surgical fistula repair experienced recurrent UTI symptoms. Nonoperative and conservative management strategies include chronic use of urinary antiseptics, intermittent antibiotics for UTI, and endoscopic clipping; however, the success of these approaches remains variable and generally limited [33,34].

In comparing our case presentations to national data, both patients were younger than the median age of 65 years identified in the NIS cohort. While our NIS analysis found no statistically significant differences in in-hospital mortality, wound complications, pneumonia, or sepsis/shock between open surgery and MIS, the confidence intervals for several outcomes, particularly hospital mortality, were wide. This indicates substantial uncertainty regarding the true effect size; therefore, these findings should not be interpreted as confirming clinical equivalence between the two approaches. Rather, they should be understood as an absence of statistically detectable difference within the limitations of our dataset, sample size, and available variables.

Postoperative sepsis/shock was the most frequent complication in both groups. Given the possibility of type II error, particularly for less frequent outcomes, further investigation involving larger or prospective cohorts is warranted to determine whether true differences exist. These findings were reflected in the second case, where the patient experienced a complicated postoperative course involving abscess formation, which required treatment with inpatient intravenous antibiotics and drainage. Notably, the length of hospital stay was significantly shorter in the MIS group than in the open surgery group (6.9 versus 7.3 days; *p* < 0.001) (Table 1).

The NIS, which is part of the Healthcare Cost and Utilization Project, represents the largest all-payer inpatient database in the United States and provides weighted estimates from a 20% stratified sample of discharges from community hospitals [35,36]. This allows for insights regarding national surgical outcomes for colovesical fistula repair.

Our findings support the current literature, which indicates that MIS approaches—including laparoscopy and robotics—have been used and are safe and effective for managing colovesical fistulas [3,6,37,38,39,40]. However, this should be interpreted cautiously, as our study design cannot rule out clinically meaningful differences that were undetected. They offer comparable short-term outcomes to open surgery in our dataset, with the added benefit of shorter length of hospital stays [39]. Given the complexity of some presentations, particularly those involving multiple fistulas, a multidisciplinary and staged approach remains critical in achieving optimal results for these specific cases [1,26].

Future applications of this research can be applied to similar patient populations when discussing the risks and benefits of open surgery versus minimally invasive repair. Emphasizing that “no statistically significant difference” is not synonymous with “equivalent” is important, and prospective randomized controlled trials are required to formally test equivalence or noninferiority. These data confirm that patients can do well with minimally invasive colovesical fistula repairs, and this approach may be considered preferential when feasible. Propensity-matched data cannot be compared to the results obtained from randomized control trials; therefore, future efforts should compare other variables that were not included in this study [41]. Future research initiatives involving large patient samples that compare open surgery and MIS techniques should be considered. Further studies could evaluate operative time, costs, and patient-reported outcomes, and explore robotic versus laparoscopic approaches, with sufficient power to detect differences in rare but clinically important complications.

This study has some limitations. First, the retrospective nature of the database analysis may introduce selection bias. Second, reliance on administrative coding in the NIS dataset limits the granularity of clinical detail and may introduce potential coding inaccuracies. Coding accuracy in administrative datasets has long been recognized as variable [42]. Although the ICD-10 codes used were selected to identify EVF cases, miscoding or variation in coding practices between institutions could result in under- or over-identification of true cases. While no validation studies exist specifically for EVF, recent research on comorbidity coding in ICD-10 demonstrates variable sensitivity ranging from 39.6% to 89.7% across conditions, with specificity generally exceeding 90% [43]. Similarly, ICD-10 coding for sepsis has shown low sensitivity (median 42.4%) but significantly high specificity (median 98.5%) [44], suggesting that administrative data substantially under-report true cases while being reliable when codes are present. Such misclassification could affect case ascertainment and outcome estimates. Third, propensity score matching adjusts for only variables included in the model, and important unmeasured clinical factors may have been unavailable or unaccounted for. Fourth, findings derived from national inpatient data may not reflect institutional practice patterns or long-term outcomes. Lastly, the inclusion of case reports may introduce bias into our findings.

## 6. Conclusions

This study combined two case reports and a retrospective national cohort analysis to evaluate the surgical management of colovesical fistulas. Minimally invasive repair successfully led to symptom resolution without recurrence during follow-up in both cases, although one patient required staged management for a complex postoperative course. In the national propensity score-matched cohort, MIS was associated with a shorter length of hospital stay than open surgery, with no statistically significant differences in major postoperative complications.

These findings align with the prior literature indicating that MIS techniques are safe and effective for colovesical fistula repair and can provide short-term outcomes similar to open surgery with the added benefit of faster recovery. Complex or multi-tract fistulas may still require staged procedures and multidisciplinary care. Recurrent UTIs that are unresponsive to antibiotics should raise suspicion for a colovesical fistula, particularly in patients with pneumaturia and fecaluria. Colonoscopy and cystoscopy remain important to exclude malignancy. Surgical management typically involves fistula takedown and segmental colon resection, with nonoperative approaches reserved for patients who are not surgical candidates. Future multicenter prospective studies should confirm these results and assess long-term outcomes, operative time, cost, and patient-reported measures.

## Figures and Tables

**Table 1:** Comparison of postoperative outcomes between patients undergoing open surgery and those undergoing MIS.

Postoperative Outcomes	Open Surgery (*n* = 1378)	MIS (*n* = 1378)	Adjusted OR	95% CI	*p*-Value
Hospital mortality, no. (%)	NR	NR	1.087	0.164–7.207	0.931
Wound complications, no. (%)	19 (1.4)	14 (1.0)	0.799	0.380–1.680	0.554
Postoperative pneumonia, no. (%)	19 (1.4)	21 (1.5)	1.249	0.627–2.487	0.528
Postoperative sepsis/shock, no. (%)	98 (7.1)	77 (5.6)	0.74	0.509–1.075	0.114
Postoperative hemorrhagic shock, no. (%)	14 (1.0)	15 (1.1)	1.065	0.500–2.268	0.87
Length of stay, mean (SD), days	7.49 (7.3)	6.89 (6.9)	1.11	1.06–1.15	<0.001 *
Total hospital charges, mean (USD)	129,102	124,672	0.995	0.95–1.04	0.82
Hospital bed size, no. (%)					0.026
Large	681 (49.4)	657 (47.7)	-	-	
Medium	398 (28.9)	461 (33.5)	1.108	0.906–1.357	0.318
Small	299 (21.7)	260 (18.9)	1.325	1.068–1.644	0.011

Abbreviations: MIS, minimally invasive surgery; OR, odds ratio; CI, confidence interval; NR, nonreportable; SD, standard deviation; USD, United States dollars; no.: number. * Statistically significant at *p* < 0.05.

## Data Availability

The data supporting the findings of this study are publicly available from the HCUP NIS database (Data Year 2016–2022; Agency for Healthcare Research and Quality, Rockville, MD, USA). Data access instructions and documentation are available at https://hcup-us.ahrq.gov/tech_assist/centdist.jsp (accessed on 21 July 2025).

## Abbreviations

The following abbreviations are used in this manuscript:

CT	Computed tomography
EVF	Enterovesical fistula
HPF	High-power field
IRB	Institutional Review Board
MIS	Minimally invasive surgery
NIS	National Inpatient Sample
CI	Confidence Interval
OR	Odds ratio
POD	Postoperative day
RBC	Red blood cell
SD	Standard deviation
SPSS	Statistical Package for the Social Sciences
UTI	Urinary tract infection
WBC	White blood cells

## Appendix A

**Table A1:** Predictors of undergoing minimally invasive surgery (MIS) versus open surgery for colovesical fistula.

Predictors of MIS	Open Surgery (*n* = 1378)	MIS (*n* = 1387)	Adjusted OR	95% CI	*p*-Value
Age at Admission, years (mean ± SD)	62.92 ± 14.03	63.02 ± 13.66	1.003	0.996–1.010	0.439
Median Age	65	65			
Admission on Weekend					
Yes, no. (%)	55 (4)	62 (4.5)	1.178	0.792–1.752	0.419
No, no. (%)	1323 (96)	1316 (95.5)	-	-	
Elective Admission					
Elective, no. (%)	1089 (79.0)	1089 (79.0)	0.969	0.787–1.193	0.768
Nonelective, no. (%)	289 (21.0)	289 (21.0)	-	-	
Sex					
Male, no. (%)	846 (61.4)	824 (59.8)			
Female, no. (%)	532 (38.6)	554 (40.2)	1.079	0.918–1.268	0.357
Race					
White, no. (%)	1033 (75.0)	1045 (75.8)	-	-	
Black, no. (%)	111 (8.1)	102 (7.4)	0.882	0.656–1.187	0.408
Hispanic, no. (%)	164 (11.9)	170 (12.3)	1.025	0.800–1.314	0.846
Asian/PI, no. (%)	16 (1.2)	18 (1.3)	1.155	0.583–2.290	0.68
Native American, no. (%)	12 (0.9)	NR	0.601	0.233–1.548	0.292
Other, no. (%)	42 (3)	36 (2.6)	0.815	0.514–1.293	0.386
Transfer Status					0.569
Not transferred, no. (%)	1347 (97.8)	1354 (98.3)	-	-	
From acute care, no. (%)	15 (1.1)	13 (0.9)	0.829	0.388–1.774	0.629
From another care facility, no. (%)	16 (1.2)	11 (0.8)	0.684	0.313–1.493	0.34
Primary Insurance					0.725
Government, no. (%)	805 (58.4)	781 (56.7)	-	-	
Private, no. (%)	520 (37.7)	537 (39.0)	1.092	0.903–1.320	0.363
Self-pay, no. (%)	30 (2.2)	34 (2.5)	1.19	0.703–2.014	0.518
Other, no. (%)	23 (1.7)	26 (1.2)	1.227	0.687–2.190	0.49
Patient Location					0.351
Nonmetro, no. (%)	52 (3.8)	73(5.3)	-	-	
Central counties of metro > 1 M, no. (%)	396 (28.7)	399 (29.0)	1.025	0.833–1.260	0.817
Fringe counties of metro > 1 M, no. (%)	380 (27.6)	397 (28.8)	0.919	0.736–1.147	0.457
Metro 250 K–999 K, no. (%)	307 (22.3)	290 (21)	0.894	0.665–1.204	0.461
Metro < 250 K, no. (%)	107 (7.8)	151 (11.0)	0.971	0.668–1.410	0.876
Micropolitan, no. (%)	106 (7.7)	98 (7.1)	1.432	0.945–2.171	0.09
ZIP Code Income Quartile					0.816
0–25%—no. (%)	266 (19.3)	281 (20.4)	0.909	0.721–1.148	0.424
26–50%—no. (%)	346 (25.1)	330 (23.9)	0.912	0.722–1.153	0.441
51–75%—no. (%)	390 (28.3)	374 (27.1)	0.964	0.749–1.239	0.772
76–100%—no. (%)	376 (27.3)	393 (28.5)	-	-	
Hospital Type					0.887
Urban teaching, no. (%)	1027 (74.5)	1032 (74.9)	-	-	
Rural, no. (%)	71 (5.2)	69 (5.0)	1.065	0.681–1.667	0.783
Urban nonteaching, no. (%)	280 (20.3)	277 (20.1)	0.995	0.718–1.672	0.673
Hospital Ownership					
Private, no. (%)	1248 (90.6)	1248 (90.6)	-	-	
Government, no. (%)	130 (9.4)	130 (9.4)	0.986	0.758–1.282	0.916
Intestinal and Bladder Malignancy					
No, no. (%)	1340 (97.2)	1340 (97.2)	-	-	
Yes, no. (%)	38 (2.8)	38 (2.8)	1.094	0.683–1.752	0.708
Elixhauser Score					
Mean ± SD (Median)	4.42 ± 7.61 (1)	4.05 ± 7.63 (0)	0.992	0.981–1.003	0.135

Abbreviations: MIS, minimally invasive surgery; OR, odds ratio; CI, confidence interval; NR, nonreportable; SD, standard deviation; M, million; K, thousand; no.: number. Data are presented as numbers (%) for categorical variables and means ± SD for continuous variables.

## Appendix B

**Table A2:** ICD-10 codes for enterovesical fistula.

Variable	ICD-10 Codes
Enterovesical Fistula	0DQ80ZZ 0DQ83ZZ 0DQ84ZZ 0DQ87ZZ 0DQ88ZZ 0DQB0ZZ 0DQB3ZZ 0DQB4ZZ 0DQB7ZZ 0DQB8ZZ 0DQE0ZZ 0DQE3ZZ 0DQE4ZZ 0DQE7ZZ 0DQE8ZZ 0DQN0ZZ 0DQN3ZZ 0DQN4ZZ 0DQN7ZZ 0DQN8ZZ 0DQP0ZZ 0DQP3ZZ 0DQP4ZZ 0DQP7ZZ 0DQP8ZZ 0TQB0ZZ 0TQB3ZZ 0TQB4ZZ 0TQB7ZZ 0TQB8ZZ

Abbreviations: ICD-10, International Classification of Diseases, 10th Revision.
